# The role of the ubiquitin proteasome system in glioma: analysis emphasizing the main molecular players and therapeutic strategies identified in glioblastoma multiforme.

**DOI:** 10.1007/s12035-021-02339-4

**Published:** 2021-03-04

**Authors:** Semer Maksoud

**Affiliations:** Experimental Therapeutics and Molecular Imaging Unit, Department of Neurology, Neuro-Oncology Division, Massachusetts General Hospital, Harvard Medical School, Boston, Massachusetts 02114, United States.

**Keywords:** Glioma, ubiquitin, proteasome, E3 ligase, deubiquitinases, PROTACs

## Abstract

Gliomas constitute the most frequent tumors of brain. High-grade gliomas are characterized by a poor prognosis caused by a set of attributes making treatment difficult, such as heterogeneity and cell infiltration. Additionally, there is a subgroup of glioma cells with properties similar to stem cells responsible for tumor recurrence after treatment. Since proteasomal degradation regulates multiple cellular processes, any mutation causing disturbances in the function or expression of its elements can lead to various disorders such as cancer. Several studies have focused on protein degradation modulation as a mechanism of glioma control. The ubiquitin proteasome system is the main mechanism of cellular proteolysis that regulates different events, intervening in pathological processes with exacerbating or suppressive effects on diseases. This review analyzes the role of proteasomal degradation in gliomas, emphasizing the elements of this system that modulate different cellular mechanisms in tumors and discussing the potential of distinct compounds controlling brain tumorigenesis through the proteasomal pathway.

## 1. Introduction.

Gliomas represent about 60% of Central Nervous System (CNS) primary tumors. Glioblastoma multiforme (GBM), the most aggressive form of brain tumor in adults, encompasses more than 54% of gliomas with an average survival hardly exceeding 15 months [1]. One of GBM hallmarks is the diffusive invasion of tumor cells in the surrounding regions of the brain, with individual infiltrating cells scattered throughout the brain parenchyma, which complicates treatment [2]. Among other properties of the tumor that also hinder its treatment is cell heterogeneity and the presence of glioma stem-like cells (GSCs), a cell subset with significant capacity for expansion and ability to generate new tumors [3].

Through multiple genomic profile studies, many genes have been sequenced in numerous tumor samples revealing the complex genetic profile of GBM, highlighting three signaling pathways commonly altered: the p53, tyrosine kinase receptor (RTK)/RAS/phosphoinositide 3-kinase (PI3K), and retinoblastoma (RB) pathways. These alterations result in uncontrolled proliferation, cell infiltration, and resistance to apoptosis [4]. These studies have also been established with the aim of facilitating the development of more effective therapies by allowing GBM to be classified, being cataloged in at least four subgroups called proneural (PN), neural (NL), classical (CL), and mesenchymal (MES); each group has particular properties associated to molecular alterations, prognosis, and sensitivity to therapies [5]. MES subtype is the most aggressive and is related to poor prognosis compared to PN subtype, with a PN→MES transition being reported in several patients undergoing radiation and chemotherapy [6].

Current standard therapy for GBM includes surgical resection, followed by radiation and co-administration of Temozolomide (TMZ), an oral alkylating agent; this therapeutic option has limited effectiveness. Surgical procedures, although represent the most effective way to increase survival of GBM patients, depend considerably on tumor location and infiltration extent [7]. Besides, high doses of radiation cannot be given due to the damage it can cause to the brain [8]. For its part, the TMZ effect, which consists of blocking the cell cycle in the G2/M phase and eventually activating apoptosis through methylation of adenine and guanine residues, is often hampered by DNA repair systems in which the role of O^6^-methylguanine methyltransferase (MGMT) is crucial [9]. However, different investigations have pointed to the development of new strategies that can improve the efficiency of treatment, such as the use of nanoparticles [10], immunotherapy [11], oncolytic viruses [12] or compounds with synergistic effects to TMZ [13].

## 2. The ubiquitin proteasome system.

Ubiquitination consists of a three-step enzymatic cascade in which proteins are labeled for degradation by the proteasome. Ubiquitin (Ub), which has a central role in this process, has 76 highly conserved amino acids and is present in all cell types, from yeasts to humans. The ubiquitin proteasome system (UPS) participates in signal transduction, cell cycle, transcription, and apoptosis events, among others. First, in an ATP-dependent process, Ub is activated by binding to a ubiquitin activating enzyme (E1), which forms a thioester bond between a cysteine residue of its active site and the carboxyl terminus of Ub. Next, Ub is subsequently transferred to a second protein called ubiquitin conjugating enzyme (E2), by transferring the thioester bond to a cysteine residue. Finally, Ub is coupled to a target protein through a peptide bond by the action of a third enzyme, termed ubiquitin ligase (E3), which is responsible for selective recognition of appropriate substrate proteins. The catalytic and substrate recruitment domains of E3 ligases can be present in a single protein, as in the case of c-CBL, or in separate subunits assembled in a multiprotein complex, such as cullin-RING ligases; in these complexes, the adapter proteins are responsible for the recruitment of substrates [14]. Given the importance of ubiquitination in the regulation of various cellular processes, it is essential that it can be reversible; this is achieved by deubiquitinase enzymes (DUBs) [15, 16].

The proteasome is a multiprotein complex responsible for degradation of most intracellular proteins. The specificity of the signal is determined by the length and structure of poly-Ub chains. However, it should be noted that there is also an Ub-independent proteasomal degradation in which auxiliary molecules or specific motifs of target proteins cooperate [17]. This multiprotein complex consists of two structural and functional parts: the catalytic core (20S proteasome) and the regulatory particles that, when united, give rise to the 26S proteasome. It has three types of proteolytic activity: similar to trypsin, chymotrypsin, and caspase (cleavage after positive, aromatic, and negative amino acids, respectively) [18]. The 19S regulatory particles, included in 26S proteasomes, are responsible for identifying, binding, deubiquitinating, unfolding, and translocating substrates into the core proteolytic chamber [19].

## 3. UPS and glioma.

The interest in UPS in the brain began mainly with observations emphasizing that Ub or UPS-related proteins were part of protein deposits in several neurodegenerative diseases, such as Alzheimer’s or Parkinson’s. Nevertheless, subsequent investigations positioned UPS in very important non-degenerative processes including growth, development, survival, synaptic function, and plasticity of neurons *(reviewed by Yi and Ehlers* [20] *and Lehman* [21]).

**Fig. 1:**
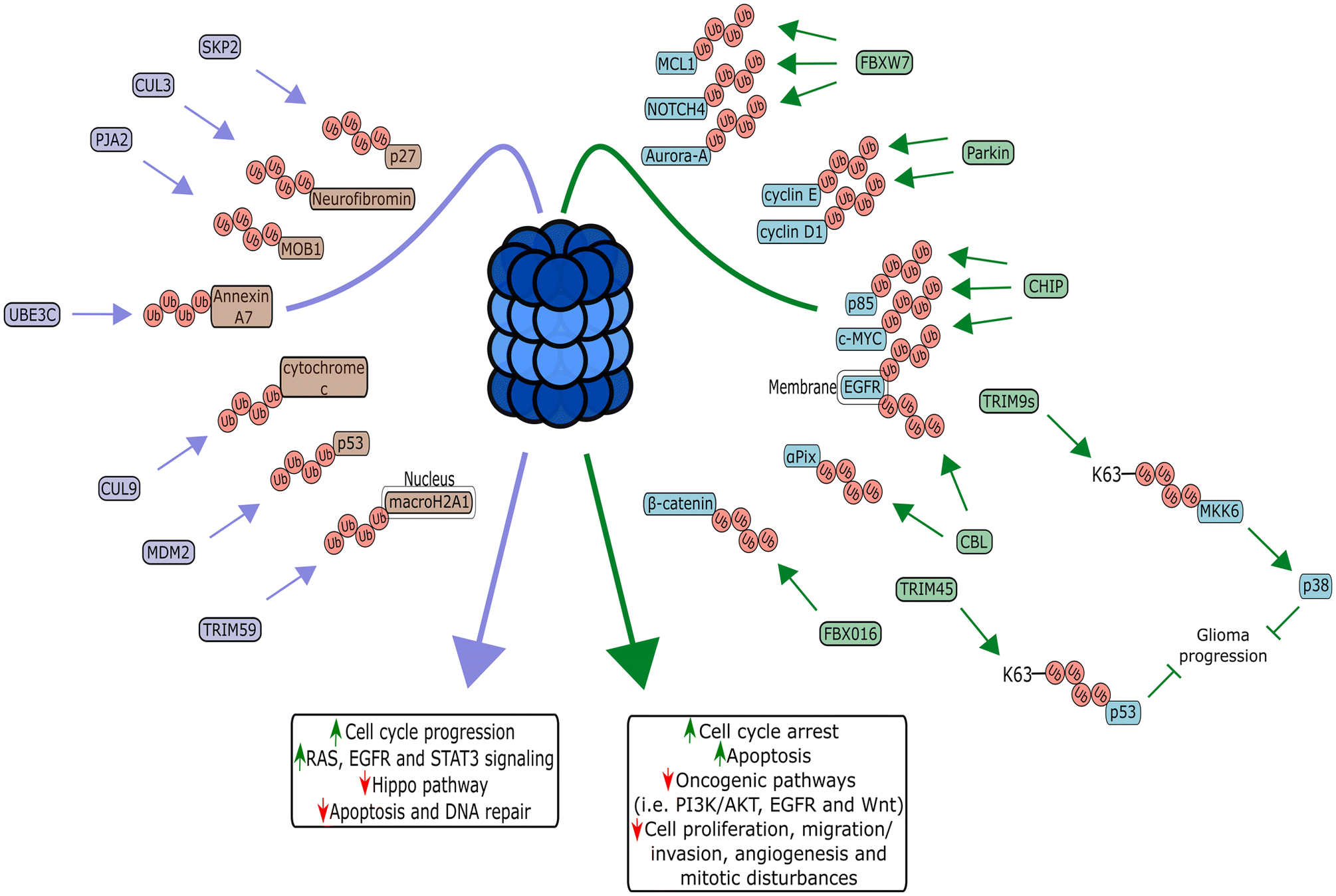
Oncological/suppressive E3 ligases identified in gliomas. Oncological and suppressor E3 ligases are designated by the colors purple and green, respectively.

Since the UPS intervenes in several cellular functions, any functional mutation or abnormal expression of its elements can lead to various disorders, such as cancer, neurodegenerative diseases, and immune disturbances; ubiquitination functions are not limited only to proteolysis but also to protein assembly, cell signaling, DNA repair, among others [22]. In cancer, ubiquitination causes activation or deactivation of tumorigenic pathways; in a siRNA screening analysis that identified relevant genes for GBM survival, 22% (12/55) were components of the 20S and 26S proteasome subunits [23]. This review discusses how different elements of the UPS regulate the suppression/progression of gliomas, highlighting genes and proteins involved and describing those investigations that have used UPS as a way of treating this type of tumor. Figure 1 indicates the suppressive/oncological E3 ligases identified in gliomas. The full names corresponding to the abbreviations used are listed in Table 1.

### 3.1. Ubiquitin conjugating enzymes.

#### 3.1.1. UBE2C/UBCH10.

In search of possible tools that can be used as diagnostic markers, attention has been given to the *UBCH10* gene, which encodes a protein belonging to the E2 family known as Ubiquitin-conjugating enzyme E2C (UBE2C/UBCH10), regulating the cell cycle in different types of carcinomas by UPS modulation, including GBM [24]. UBCH10 expression has been evaluated in normal brain, gliosis, grade II astrocytic tumors, and GBM, revealing a direct correlation between this expression and the histological grade of tumors [25, 26]. The expression of this enzyme is also linked to poor prognosis and resistance to therapy in patients [27, 28]. In addition, the analysis of UBCH10 expression allowed for differentiation of tumor tissue from gliotic or normal tissue [25]. UBCH10 would facilitate the formation of CDK1-cyclin B1 complexes that initiate mitosis [24]; thus, this enzyme could not only be used as a diagnostic marker but also as a therapeutic target. Its knockdown caused the inhibition of proliferation, activation of p53, and Bax-dependent apoptosis in U251 glioma cells [29].

#### 3.1.2. UBE2S.

The ubiquitin conjugating enzyme E2S (UBE2S) is commonly overexpressed in grade III and IV gliomas, being phosphorylated by AKT, which prevents its proteasomal degradation. UBE2S increased expression in GBM is related to poor prognosis and low sensitivity to chemoradiotherapy [30]. In approximately 40–60% of GBM, the tumor suppressor phosphatase and tensin homologue deleted on chromosome 10 (PTEN) is mutated [31], where the loss of its function leads to constitutive activation of the PI3K/AKT/mTOR signaling pathway [32]. In GBM, UBE2S binds to several components of the non-homologous end-joining (NHEJ) complex, cooperating in the repair of DNA double stranded breaks (DSBs) that can be caused by internal metabolites, including reactive oxygen species (ROS) or external factors like ionizing radiation. *In vivo* experiments reveal that UBE2S knockdown makes GBM tumors more sensitive to etoposide, a DNA damage agent. In consequence, the AKT1/UBE2S/NHEJ axis assists in GBM chemo and radioresistance [30, 33, 34].

### 3.2. Oncological ubiquitin ligase enzymes.

#### 3.2.1. SCF^SKP2^.

The first investigation involving UPS in glioma development was described by *Piva et al*. [35]. In their study they revealed, using the proteasome inhibitor LLnL, that proteasomal degradation of p27 would be one of the causes for malignant transformation of gliomas. Previously, they showed that p27 levels in astrocytic tumors were reduced, while in GBM were almost nil [36]. The G1/S transition of the cell cycle is regulated by p27/KIP1 through inhibition of cyclin D-CDK4 and cyclin A/E-CDK2 [37]; the progression through the cell cycle is promoted by cyclin-dependent kinases (CDKs) action.

SKP1, CUL1, and F-box (SCF) complexes are a class of E3 enzymes that ensure the specific recognition and ubiquitination of different substrates through various F-box proteins [38]. S-phase kinase-associated protein 2 (SKP2) is required for G1/S transition by facilitating ubiquitination and subsequent degradation of p27/KIP1 [39]. There is an inverse correlation between p27/KIP1 and SKP2 in a series of astrocytic gliomas: SKP2 expression was absent or greatly reduced in well-differentiated astrocytomas, but increased significantly in several GBM samples [40]. PTEN has been determined to increase protein stability of p27/KIP1 by reducing SKP2 levels [41]: there is the possibility that *PTEN* genetic alterations, which are common in GBM, are responsible for increased and reduced levels of SKP2 and p27/KIP1, respectively [40]. In addition, since SKP2 is overexpressed in several types of tumors and regulates the proteasomal stabilization/degradation of relevant proteins in glioma tumorigenesis, such as p21, myelocytomatosis (MYC) or cyclin D1, it could also be considered as a therapeutic target; several SKP2 inhibitors have been developed with beneficial effects *in vitro* and *in vivo* [42–45].

#### 3.2.2. CUL3.

The Cullin-3 enzyme (CUL3) is part of the Cullin-RING E3 ligase complexes, which, together with the adapter protein KBTBD7, induces the ubiquitination and degradation of neurofibromin in response to growth factors [46]. The neurofibromin tumor suppressor is a known RAS GTPase activating protein (RasGAP) generally mutated in various types of tumors, including GBM [47]. One of the signaling mechanisms frequently altered in different types of human cancer is the RAS pathway, either by mutations of *RAS* or in genes encoding RasGAPs, a series of negative regulators hydrolyzing RAS-GTP, causing hyperactivation of the pathway [48]. Mitigation of the anticancer activity of neurofibromin could take place not only by genetic mutations but also by proteasomal degradation, as in other tumor suppressors, such as p53 and PTEN [47]. CUL3 knockdown stabilizes neurofibromin, inactivating the RAS pathway and inhibiting GBM cell proliferation [46].

#### 3.2.3. PJA2.

Another E3 ligase exerting oncogenic function is Praja2 (PJA2), expressed in multiple tissues and cells, including the brain [49]. In human glioma cells, this enzyme binds and ubiquitin the tumor suppressor Mps one binder 1 (MOB1), a component of large tumor suppressor kinases 1/2 (LATS1/2) involved in the Hippo pathway. The inverse correlation between PJA2 and MOB1 was also demonstrated *in vivo*. MOB1 proteasomal degradation and the consequent attenuation of the Hippo signal drive GBM growth. There is a direct correlation between PJA2 expression and glioma aggressiveness, so it could be considered as a prognostic marker [50].

#### 3.2.4. UBE3C.

Little is known about substrates and the implications that Ubiquitin-protein ligase E3C (UBE3C) action may have on cells. In the particular case of gliomas, UBE3C upregulation was found in glioma tissues compared to surrounding normal tissues; its overexpression promoted invasion and mobility of GBM cells. Additionally, UBE3C could function as a correct prognosis biomarker, since higher levels of UBE3C expression were detected in patients with aggressive clinicopathological characteristics, such as high tumor grade, metastasis, and poor differentiation. Regarding its mechanism of action, UBE3C induces the proteasomal degradation of Annexin A7 [51]: this protein is believed to act as a tumor suppressor in GBM via attenuation of the epidermal growth factor receptor (EGFR) signaling [52].

#### 3.2.5. PARC/CUL9.

One of the key events of the intrinsic apoptotic pathway is the permeabilization of the mitochondrial membrane, deriving in the release of proapoptotic factors from this organelle, including cytochrome c, which will be assembled in the caspase activating apoptosome [53]. Nevertheless, in glioma cells, a control mechanism for cytochrome c was identified. It was determined that Parkin-like cytoplasmic protein (PARC/CUL9) is an E3 ligase implicated in the ubiquitination and subsequent proteasomal degradation of cytochrome c, a process that does not occur in normal dividing cells. Low levels of APAF-1, the main protein associated with cytochrome c after its release, would be responsible for its degradation [54].

#### 3.2.6. MDM2.

The protein p53 is a tumor suppressor that acts as a sensor of cell stress whose activation often initiates apoptosis, cell cycle arrest, and DNA repair [55]. Under normal conditions, p53 protein levels are usually low and controlled by the proteasome, being mouse double minute 2 homolog (MDM2) one of the E3 ligases binding to this protein [56]. In primary GBMs, the inactivation of the p53 pathway is frequent due to MDM2 overexpression. On the other hand, p53 mutations are more common as the histological grade of gliomas increases, suggesting that this protein plays a role in the generation of secondary GBMs [57].

Likewise, the MDM2-p53 interaction is regulated by several mechanisms. For example, positive feedback between p53 and PTEN has been reported, where the latter blocks p53 degradation and this induces PTEN expression [58]. *Kim et al*. [59] reported that Merlin, a tumor suppressor related to neurofibromatosis-2, stabilizes p53 levels by inducing MDM2 degradation in glioma cells. Additionally, *Park et al*. [60] identified a complex which dephosphorylates p53 known as GAS41-PP2Cβ, generally amplified in human gliomas [61]; p53 phosphorylation blocks its interaction with MDM2. Other posttranslational modifications inhibiting MDM2-p53 interaction are acetylation, sumoylation and neddylation [55]. Targeting the MDM2-p53 axis could give us positive results in the search for new therapies: the construction of a mutant p53 protein with substitution of amino acid residues avoiding its ubiquitination inhibited glioma cell proliferation *in vitro* [62].

#### 3.2.7. TRIM59.

One of the mechanisms of EGFR-driven tumorigenicity in gliomas involves the E3 ligase Tripartite motif family (TRIM) 59. CDK5 is activated in EGFR signaling, which has been linked to poor prognosis in GBM patients and to *in vitro* self-renewal of GSCs [63]. CDK5 phosphorylates TRIM59, leading to its nuclear translocation where it will initiate the ubiquitination and degradation of macroH2A1, a tumor-suppressive histone; this eventually results in increased STAT3 signaling and glioma tumorigenicity [64]. TRIM59 also exerts an oncological effect on gliomas through a mechanism not dependent on its E3 ligase activity [65].

### 3.3. Suppressive ubiquitin ligase enzymes.

#### 3.3.1. FBXW7.

Those responsible for the ubiquitination of proteins through SCF complexes are F-box proteins, which represent the variable component of these complexes. *FBXW7* encodes one of the more than 70 F-box proteins identified in humans. Unlike other F-box proteins involved in the ubiquitination of positive and negative regulators of the cell cycle, all known SCF^Fbxw7^ targets are promoters of cell proliferation [66, 67]. *FBXW7* is mutated in different cancer cell lines and human tumors, including gliomas [68]. A known substrate of this anti-tumor ligase is mTOR, whose signaling promotes cell survival, proliferation, and motility [69]. Besides, FBXW7 [70], among other ligases, modulates the proteasomal degradation of MYC, an oncological transcriptional factor generally overexpressed in gliomas [71].

FBXW7 expression is reduced in GBM and this has been correlated to lower survival in patients. In protein extracts from biopsies of GBM samples, it was found that FBXW7 loss of function resulted in the accumulation of Aurora-A and NOTCH4 [67]. Aurora-A overexpression, a protein required for G2/M transition, causes centrosome amplification and cytokinesis defects that generate abnormal cells with a high probability of undergoing a malignant transformation [72]. Little is known about the role of NOTCH4 in cancer, but it is probably linked to blood vessel formation in tumors [73]. Furthermore, *FBXW7* overexpression is involved in proliferation inhibition of glioma cells, while its deletion causes instability in chromosome segregation during mitosis, a process controlled by several SCF^Fbxw7^ targets, including Aurora-A [67].

Also, *Lin et al*. [74] reported that overexpression of this ligase makes GBM cells more sensitive to the TMZ effect, causing an increase in apoptosis, cell cycle arrest at the G2/M transition, and a decrease in cell migration by downregulation of MCL-1, Aurora-B, and NOTCH1, respectively. Interestingly, *Yang et al*. [75] identified a circular RNA encoding circ-FBXW7, whose expression in GBM samples was lower compared to the surrounding normal tissue, being capable of antagonizing the stabilization of the oncoprotein c-MYC initiated by the deubiquitinase USP28; circ-FBXW7 expression was linked to greater survival in patients.

#### 3.3.2. Parkin.

Parkin is an E3 ligase encoded by the *PARK2* gene, which is usually mutated in different types of cancer, including gliomas. Parkin activity reduction causes accumulation of cyclin E and D1, producing mitotic disturbances. The induction of Parkin expression, generally low in GBM, generates a blockade of the cell cycle in the G1 phase, decreasing glioma cell proliferation *in vitro* and *in vivo*. The expression of this ligase is correlated with greater survival and a lower degree of malignancy in GBM patients [76, 77]. Further, its overexpression in GBM cells mitigated metastasis, cell invasion, and the epithelial-mesenchymal transition (EMT), albeit through a proteasome-independent mechanism [78].

#### 3.3.3. CHIP.

A component of the UPS with anticancer activity is the E3 ligase called carboxyl terminus of Hsc70-interacting protein (CHIP), which connects substrates of chaperones such as the heat shock protein 90 (HSP90) with the proteasome [79]. Nevertheless, some evidence also indicates that it has oncogenic properties in gliomas [80, 81]; this may depend on the type of cell and the presence of proteins that can interact with it. CHIP induces the ubiquitination and degradation of the oncoprotein c-MYC in glioma cells [82]; its knockdown magnifies the metastatic properties of these cells. In different types of tumors, c-MYC is commonly overexpressed, contributing to uncontrolled cell proliferation [83]. In fact, CHIP mRNA levels are lower in GBM compared to normal tissues [82]. Hence, CHIP could be considered as a target for the treatment of tumors where c-MYC has an active role.

In addition, CHIP forms an E3 ligase complex with HSP70 and p42 in glioma cells [84]: the latter is an isoform of the EBP1 protein that has anticancer activity, unlike another isoform of the same protein known as p48, which is oncological [85]. This complex is capable of causing the proteasomal degradation of p85, a subunit of PI3K; excessive activation of the PI3K/AKT signaling pathway is common in several types of cancer [86]. Co-expression of CHIP/HSP70 and p42 decreased p48 levels and markedly inhibited glioma growth in mice [84].

Another known CHIP substrate is EGFR, a transmembrane protein with tyrosine kinase activity frequently overexpressed in GBM [87, 88]. EGFR phosphorylation activates the PI3K/AKT, MAPK, and SRC signaling pathways involved in cell proliferation, metastasis, and survival [89]. However, a negative CHIP regulator known as CSN6 has been identified in GBM, a constitutive photomorphogenesis 9 (COP9) signalosome (CSN) subunit with oncogenic properties [90], whose expression levels are significantly higher in GBM tumors in contrast to normal brain tissues, as well as in glioma cell lines. CSN6 promotes GBM proliferation, migration, invasion, and tumorigenesis through upregulation of EGFR by blocking its ubiquitination; this happens as a result of interactions with CHIP that cause its degradation, although CHIP auto-ubiquitination occurs through an unknown mechanism [91]. Accordingly, the EGFR/CHIP/CSN6 pathway should be explored in more depth if it is desired to use CHIP as a therapeutic approach in GBM.

#### 3.3.4. CBL.

It is believed that the RING-type E3 ubiquitin ligase named c-CBL has a role as a tumor suppressor by inducing proteasomal degradation of proteins involved in cell proliferation and migration, such as paxillin, FAK, and EGFR [92]. In addition to EGFR, other tyrosine kinase receptors are usually overexpressed in pediatric high-grade gliomas, including platelet-derived growth factor receptors (PDGFRs), vascular endothelial growth factor receptor (VEGFR), among others, are targeted by CBL [93–95]. In particular, *EGFR* gene amplification has been detected in approximately ~ 40% of gliomas [57], as well as frequent mutations that generate a receptor unable to recruit this ubiquitin ligase [96]: this leads to less receptor internalization and degradation, with the signaling resulting from EGFR dimerization being an aggravating factor in gliomas [45, 97].

Among other substrates of c-CBL is αPix, a guanine nucleotide exchange factor that activates the Rho family and is involved in migration, angiogenesis, and cell propagation. In A172 (human) and C6 (rat) glioma cells, c-CBL is not expressed, causing αPix accumulation and consequently promoting cell migration and invasion; this in contrast to other cell lines that do express c-CBL [98]. Nonetheless, other GBM cell lines do not express αPix and remain highly invasive, so other c-CBL/αPix-independent mechanisms operate. These observations would explain why αPix levels are higher in tissues of GBM patients [99], due to the apparent silencing of c-CBL expression.

Future investigations should analyze the mechanisms of silencing or attenuation of the expression of suppressive E3 ligases. Indeed, *Seong et al*. [100] demonstrated that *c-CBL* exon skipping occurred in A172 and C6 glioma cells, as well as in brain tissues of GBM patients, generating two isoforms of the protein which were rapidly degraded by the proteasome. Interestingly, the *c-CBL* exon skipping happens when cells grow in high density or under hypoxic conditions, suggesting that environmental factors activate *trans* elements catalyzing the exon skipping.

#### 3.3.5. TRIM9.

Ubiquitination is generally related to proteasomal degradation, but it is more complex since it regulates several cellular processes: this depends on the length of the ubiquitin chains and the compromised lysine residues. Recently, *Liu et al*. [101] determined that a short isoform of TRIM9, known as TRIM9s, promotes K63-linked poly-ubiquitination of MAPKK6 (MKK6) at residue Lys82, decreasing the availability of this residue for K48-linked poly-ubiquitination related to proteasomal degradation. MKK6 is known as one of several positive regulators of p38, a member of the mitogen-activated protein kinase (MAPK) family [102]: p38 is generally known as a tumor suppressor by blocking cell proliferation and activating apoptosis [103]. MKK6-p38 signaling plays a critical role in suppressing GBM progression *in vitro* and *in vivo* [101].

Interestingly, MKK6-p38 signaling stabilizes TRIM9s by blocking its K48-linked poly-ubiquitination, establishing positive feedback. Unfortunately, levels of TRIM9s are lower in GBM tissues compared to normal tissues. *Liu et al*. [101] suggest that in normal brain cells TRIM9s stabilizes MKK6 and this, in turn, activates p38 bound to TRIM9s, which phosphorylates preventing its degradation. On the contrary, in GBM cells the transcriptional downregulation of TRIM9s decreases its protein levels, generating a consequent increase in MKK6 proteasomal degradation, reduction of p38 activation, degradation of the remaining TRIM9s molecules and ultimately tumor progression.

The question remains about the mechanisms responsible for TRIM9 isoforms generation or its low expression in gliomas. In addition, another scale of complexity is added to the UPS and glioma relationship: the intervention of proteins regulating the proteasome activity depending on the type of ubiquitination initiated.

#### 3.3.6. TRIM45.

The E3 ligase TRIM45 is another member of the TRIM family functioning as a tumor suppressor. Its expression is reduced in glioma tissues [104] despite being elevated in the healthy adult brain [105]. This ligase inhibits cell proliferation and induces apoptosis in glioma cells as well as decreases tumor growth *in vivo*. TRIM45 stimulates the K63-linked poly-ubiquitination of p53 in glioma, decreasing its availability for the K48-linked poly-ubiquitination that leads to its degradation [104].

#### 3.3.7. FBXO16.

Recently, another UPS element acting as a tumor suppressor was identified. *Khan et al*. [106] found a member of the FBXO protein family known as FBXO16 interacting with β-catenin, an important element of the Wnt signaling pathway, guiding it toward its proteasomal degradation. This prevents the hyperactivation of the Wnt pathway, which drives the progression of glioma malignancy [107]. FBXO16 expression in gliomas is low, so it would be appropriate to evaluate how this expression is controlled in this type of cancer to eventually use the FBXO16 → β-catenin pathway as a therapeutic route.

### 3.4. Deubiquitinase enzymes.

Similar to E3 ligases, oncogenic and anticancer functions have been reported for deubiquitinase enzymes in gliomas. Among the oncogenic effects of deubiquitinases are the mitigation of tumor suppressors, increased apoptotic resistance, stabilization of oncoproteins, and maintenance of oncogenic transduction signals. Besides the classic effects observed by blocking/activating various elements of UPS, such as modulation of cell proliferation or apoptosis, the evidence shows that deubiquitinases also collaborate in the radio/chemoresistance development, microenvironment modulation, and maintenance of GSCs stemness (capacity for self-renewal and multipotentiality) [108]. Other studies reveal deubiquitinase enzymes participating in cell differentiation processes, including EMT *(reviewed by Suresh et al*. [109]). Table 2 summarizes the expression, mechanisms of action, and effects of deubiquitinases in gliomas.

### 3.5. UPS and glioma stem-like cells.

Cell heterogeneity represents one of the main reasons for a poor response to treatment in gliomas, highlighting the presence of GSCs, a highly tumorigenic and aggressive cell subset with a significant capacity for expansion [3]. The accumulated evidence shows that proteasome inhibitors possess anticancer activity against different types of tumors, including gliomas [144–146]. In the particular case of GSCs, several studies show the pro-apoptotic activity of these inhibitors [147–150].

*Low et al*. [151] analyzed the effect that knockdown of several E3 ligases would cause on U87MG cells and GSCs phenotype, aiming to identify those compromised in apoptosis resistance, cell cycle progression, and stemness maintenance. Although therapies are usually directed at induction of GSC apoptosis, some therapeutic approaches also seek to mitigate stemness by stimulating cell differentiation. Five ligases associated with apoptosis resistance in U87MG cells (UBE3B, CNOT4, TRIM52, TRIM13, and MARCH9) were identified. On the other hand, NFX1, TRIM41, FBXO21, FBXL20, and FBXO44 ligases are related to cell cycle progression in these differentiated cells. Interestingly, knockdown of several of these ligases did not cause apoptosis in GSCs unlike U87MG cells: in GSCs, knockdown of UBE3E3, TRIM3, TRIM52, and NFX1 ligases generated apoptosis, while deletion of TRIM41, FBXL20, RNF25, and TRIM13 produced the loss of stem cell markers, generating more differentiated phenotypes. According to the authors, it would be convenient to use differentiation therapies together with cytotoxic agents to arrest growth or eliminate cell subpopulations responsible for tumor recurrence. Differences in the activity of UPS proteins when comparing differentiated glioma cells and GSCs suggest that UPS is subjected to dynamic changes during the maintenance of stemness in GSCs and their differentiation process.

Several proteasome inhibitors develop a significantly higher cytotoxic effect on GSCs compared to their differentiated counterparts through an ATF3-dependent apoptotic process [152]. However, some clinical trials using Bortezomib, an inhibitor of the chymotrypsin-like proteasome activity, do not show equally positive results and this could be explained by the cytotoxicity conferred to GSCs but not to the bulk tumor, which apparently would not affect the total tumor mass [153, 154]. Indeed, the most differentiated cells are approximately 1000 times more resistant to proteasome inhibitors [152].

## 4. UPS targeting in glioma.

It is not known exactly why cancer cells are more sensitive to proteasome inhibitors compared to normal cells. One possible reason derives from several investigations demonstrating that proteasomal function is more active in tumors, which is important for malignant phenotype maintenance [155]. The disruption of the proteasomal function could lead to an intensification of oncoprotein effects or decrease the availability of tumor suppressors. Different proteasome inhibitors such as Lactacystin, N-acetyl-leu-leu-norleucinal, MG132, and Proteasome inhibitor II induce apoptosis in glioma cells (Table 3).

In addition to UPS-mediated protein degradation in eukaryotic cells, degradation of intracellular and extracellular proteins also takes place through autophagy [156]. *Ge et al*. [157] demonstrated that MG132 induced autophagy in glioma cells, blocking cell proliferation and activating apoptosis. What is interesting is that co-treatment of these cells with MG132 and 3-MA, an autophagy inhibitor, led to increased cell death. These observations open the possibility of considering autophagy inhibitors as therapeutic tools together with proteasome inhibitors. Nonetheless, the dual role of autophagy in cancer has also been manifested in gliomas, as it has been shown to disturb proliferation and tumorigenicity [158, 159] or to promote survival of cancer cells in stressful situations [160, 161].

Bortezomib is a drug approved by the Food and Drug Administration and European Medicine Agency for multiple myeloma treatment, validating the UPS as a therapeutic anticancer target [162]. Various mechanisms of action causing cell cycle arrest and apoptosis in GBM have been described for Bortezomib [148, 163–165]. This inhibitor is also capable of exerting a synergistic anti-glioma effect in combination with autophagy suppressants [166, 167]. Likewise, the inhibition of anti-apoptotic proteins, usually overexpressed in brain tumors and causing resistance to treatments [168], makes glioma cells more sensitive to the pro-apoptotic effect of Bortezomib [169]. Furthermore, Bortezomib downregulates MGMT levels by inhibiting NFκB activation [170, 171]; this protein has a central role in TMZ resistance [172]. Besides, NFκB is constitutively activated in GBM, boosting migration, invasion, and resistance to chemotherapy [173]; the *IκBα* gene is often deleted in tumor samples from GBM patients [174].

In addition to the classic proteasome inhibitors, different studies over the years have revealed the presence of several synthetic and natural compounds exhibiting anticancer effects in gliomas through UPS targeting. Among the synthetic compounds are different drugs used in the treatment of other diseases, such as Saquinavir (HIV), Troglitazone (diabetes), and Disulfiram (alcoholism), isopeptidase inhibitors (G5) and γ-secretase suppressors (LLNle). The natural compounds cover a wide range of products derived from bacteria (Geldanamycin), animals (Bufalin), and plants (Thymoquinone, Hypericin, Paeoniflorin, Sophoridine, Curcumin, and Obtusaquinone) with anti-glioma activity. Unfortunately, only four of the compounds listed in Table 3 (Bortezomib, Disulfiram, Hypericin, and Curcumin) have been tested in clinical trials, and none have yet entered clinical phase III; their efficacy is relatively limited, which is why the development of analogs with greater ease of penetration into the BBB and less toxicity is urgently needed.

## 5. Remarks.

Although clinical trials show that proteasome inhibitors are not significantly efficient in treating gliomas, second-generation proteasome inhibitors have been produced with better pharmacokinetic properties [203, 204]. It is hoped that in the future these new inhibitors will generate better results in the treatment of gliomas and other diseases, such is the case of the proteasome inhibitor Marizomib [205]. However, the inhibition of the proteasome would cause a non-selective blockage of the degradation of all proteins subjected to this process. Therefore, future therapeutic strategies in the context of UPS-glioma should focus on going as downstream as possible, such as targeting E3 enzymes.

Based on this suggestion, the aforementioned study carried out by *Low et al*. [151] shows different E3 ligases that could be considered as potential therapeutic targets in gliomas. Note that these ligases were not described in any of the previously mentioned E3 ligases (oncological or suppressive), which demonstrates that the participation of UPS elements in gliomas is much more complex than disclosed. The information available on the relationship between UPS and glioma, although abundant, remains incomplete. More studies are needed aiming to identify cellular elements that regulate or are being regulated by UPS, involved in some way in the development or suppression of gliomas: several elements of the UPS exhibiting oncogenic or suppressive activities have not yet been evaluated in brain tumors. In this regard, *Vlachostergios et al*. [206] compile a set of proteins participating in motility and invasion of tumors, including gliomas, whose regulation would be determined through UPS, presenting a group of potential targets many of which are overexpressed.

Additionally, it is pertinent to suggest for future studies that these should not only focus on cell and biochemical variations occurring in cancer cells but also in the tumor microenvironment. The glioma microenvironment is composed of different types of non-cancerous cells, an extracellular matrix, and unique cell subtypes (astrocytes, microglia, and neurons), which give properties that distinguish the brain from the rest of the body. For example, the evidence indicates that through different mechanisms macrophages, regulatory T lymphocytes, and neurons contribute to glioma progression, while dendritic cells and effector T lymphocytes initiate anticancer activities *(reviewed by Quail and Joyce* [207]). An intriguing topic for further study might be how the UPS could influence the tumor microenvironment modulation and what its putative effect would be in gliomas.

## 6. Future perspectives: PROTAC.

The use of drugs in therapies has certain limitations, such as the development of resistance, unwanted side effects, and/or their inefficiency in targeting proteins that lack enzymatic activity, for instance, proteins that function through protein interactions or scaffolding proteins. Proteolysis Targeting Chimeras (PROTACs) are a new and useful tool for drug designing in which target protein levels are regulated by proteasomal degradation. PROTACs have two ligands, connected through a linker, bound to a protein of interest and an E3 ligase: this way a ternary complex (PROTAC, the protein of interest, and E3 ligase) is assembled, inducing ubiquitination and proteasomal degradation of target proteins [208]. There is even a variant formed by two ligands for ligases known as Homo-PROTAC, which allows the self-degradation of E3 ligases after dimerization. This variant is very useful considering these types of enzymes have multiple domains and lack an active site, so their activities are not usually easy to suppress using conventional inhibitors [209].

The selectivity of PROTAC-linked drugs is superior [210], enabling the targeting of mutated proteins that often cannot be targeted by drugs alone [211], being useful in the context of drug resistance evasion in diseases. However, it is necessary to continue improving properties related to bioavailability, tissue distribution, toxicity, pharmacokinetics, and molecular weight of PROTACs [212], as well as the development of more stable ternary complexes [210].

In diseases like cancer, the PROTAC tool has been used for selective degradation of proteins, including kinases, transcription factors, or nuclear receptors, obtaining promising results in breast, lung, colon, lymphoma, prostate, myeloma, leukemia, among other types of cancer [213–218]. Besides, PROTAC is effective in tumor growth inhibition *in vivo* [216, 218–221], being able to reduce the proliferation or initiate apoptosis in cancer cells with a lower IC_50_ when contrasted to unconjugated drugs [216, 222]. PROTAC has not yet been used in GBM, but the results obtained in other types of cancer suggest that it could be useful in this type of tumor. The big question remains how effective would be the passage of PROTACs through the BBB. This review could work as a starting point in choosing target proteins and ligases for PROTACs design in gliomas; moreover, it presents a set of oncogenic ligases that could be used in studies employing Homo-PROTACs. This suggestion would represent a radical change in the way the UPS and glioma relationship is treated because instead of inhibiting the UPS to treat this type of tumor we would be taking advantage of it for selective degradation.

## 7. Conclusions.

Genetic or protein alterations in elements of the UPS or molecules regulating them cause accumulation of oncoproteins or degradation of tumor suppressors. This review covers those studies describing the complex relationship between UPS and glioma. This type of research is necessary as it allows to establish the basis for the creation of new treatments that are increasingly selective, efficient, and less toxic. Although in myeloma the use of Bortezomib has been very beneficial, in gliomas the administration of classic proteasome inhibitors has certain limitations; nevertheless, these compounds could be useful for adjuvant or combined therapy. However, the different reports in which classic proteasome inhibitors, synthetic, and natural compounds were used for UPS targeting have undoubtedly shown the great potential of the proteasomal degradation route in glioma regulation. This is why other approaches such as targeting more specific proteins (E3 ligases) or using PROTACs could be implemented.

## Figures and Tables

**Table 1:** Abbreviations.

Abbreviation: Full name
BBB: Blood-brain barrier.
CDKs: Cyclin-dependent kinases.
CHIP: Carboxyl terminus of Hsc70-interacting protein.
CL: Classical.
CNS: Central Nervous System.
COP9: Constitutive photomorphogenesis 9.
CSN: Signalosome.
CUL: Cullin.
DSBs: DNA double stranded breaks.
DUB: Deubiquitinase.
E1: Ubiquitin activating enzyme.
E2: Ubiquitin conjugating enzyme.
E3: Ubiquitin ligase.
EGFR: Epidermal growth factor receptor.
EMT: Epithelial-mesenchymal transition.
GBM: Glioblastoma multiforme.
GSC: Glioma stem-like cells.
HSP90: Heat shock protein 90.
LATS: Large tumor suppressor kinase.
MAPK: Mitogen-activated protein kinase.
MDM2: Mouse double minute 2 homolog.
MES: Mesenchymal.
MGMT: O6-methylguanine methyltransferase.
MKK6: MAPKK6.
MOB1: Mps one binder 1.
MYC: Myelocytomatosis.
NHEJ: Non-homologous end-joining.
NL: Neural.
PARC/Cul9: Parkin-like cytoplasmic protein.
PDGFRs: Platelet-derived growth factor receptors.
PJA2: Praja2.
PI3K: Phosphoinositide 3-kinase.
PN: Proneural.
PROTAC: Proteolysis Targeting Chimeras.
PSI: Synthetic Proteasome Inhibitor.
PTEN: Phosphatase and tensin homologue deleted on chromosome 10.
RasGAP: RAS GTPase activating protein.
RB: Retinoblastoma.
ROS: Reactive oxygen species.
RTK: Receptor tyrosine kinase.
SCF: Skp1, Cul-1 and F-box.
SKP2: S-phase kinase-associated protein 2.
TMZ: Temozolomide.
TRIM: Tripartite motif family.
Ub: Ubiquitin.
UBE2C/UBCH10: Ubiquitin-conjugating enzyme E2C.
UBE2S: Ubiquitin-conjugating enzyme E2S.
UBE3C: Ubiquitin-protein ligase E3C.
UPS: Ubiquitin Proteasome System.
VEGFR: Vascular endothelial growth factor receptor.

**Table 2:** Deubiquitinases involved in glioma suppression/exacerbation.

Deubiquitinase	Expression and Mechanism of action	Effects	References
Tumor-promoter deubiquitinases			
HAUSP	Higher expression in glioma. Stabilization of MDM2, LSD1, and NANOG.	↓Survival of patients. ↑Proliferation and invasion. ↓p53 signaling pathway. ↑Stemness of glioma cells.	[110–113]
OTUB1	Overexpression in GBM. Expression correlated with histological grade. Stabilization of Snail and Vimentin.	↓Survival of patients. ↑Migration and EMT.	[114]
USP1	Up-regulated in GBM. Stabilization of ID1, ID2, and CHEK1, regulators of the DNA damage response and stem cell maintenance. Stabilization of EZH2, a transcriptional repressor of several anticancer proteins.	↑Survival and growth of GSCs. ↑Radioresistance of GBM. ↑Survival of proneural glioma cells. ↑Proliferation of glioma cells.	[108, 115, 116]
USP3	Upregulation in GBM. Stabilization of Snail, a transcription factor promoting EMT.	↓Survival of patients. ↑Invasion, migration, and tumor growth. ↑EMT.	[117]
USP4	Upregulation in GBM. Stabilization of PCNA, Bcl-2, p-ERK1/2, and regulation of TGF-β.	↑Proliferation, TMZ resistance, and ERK pathway. ↓p53-dependent apoptosis. ↓Survival of patients.	[118, 119]
USP5	In GBM, an aberrant splicing event occurs generating an oncogenic isoform of USP5.	↑Tumorigenicity.	[120]
USP8	Stabilization of the antiapoptotic protein FLIP.	↑GBM resistance to TRAIL-induced apoptosis.	[121]
USP9X	Prevents β-catenin degradation, which promotes the expression of c-MYC and cyclin D1. Stabilization of ALDH1A3.	↑Wnt/β-catenin signaling pathway. ↑Proliferation and survival. ↑Tumorigenicity and self-renewal of GSCs.	[122, 123]
USP10	Overexpression in GBM. Mechanism of action unknown in glioma.	↓Survival of patients.	[124]
USP13	Prevents c-MYC ubiquitination induced by the ligase FBXL14.	↑GSC self-renewal and tumorigenic potential.	[125]
USP22	Increased expression in glioma samples. Stabilization of CDK1, CDK2, cyclin B1, BMI1, and KDM1.	↑Proliferation, survival, migration, and invasion of glioma cells. ↑Tumorigenesis. ↑Stem cell self-renewal. ↓Survival of patients.	[126–130]
USP28	Overexpression in glioma. Stabilization of the oncoprotein c-MYC.	↓Survival of patients. ↑Proliferation and tumorigenicity.	[131]
USP39	Overexpression in glioma. Stabilization of the oncoprotein TAZ.	↑Proliferation, migration, and invasion.	[132]
USP44	Upregulation in GBM. Stabilization of the oncoprotein Securin.	↓Apoptosis and survival of patients. ↑Proliferation, tumorigenesis, migration, and invasion.	[133]
USP48	Expression correlated with glioma malignancy. Stabilization of Gli1 with subsequent activation of the Hedgehog signaling pathway.	↑Proliferation and tumorigenicity.	[134]
**Tumor-suppressor deubiquitinases**			
USP2a	Conflicting results. It is overexpressed in glioma tissues and its levels correlate with an increase of the tumor histological grade. However, it stabilizes the levels of the pro-apoptotic protein Mdm4.	↑p53-dependent intrinsic apoptosis in GBM.	[135–137]
USP11	Inhibits the ubiquitination and proteasomal degradation of the protein PML, an essential component of nuclear structures.	↓Proliferation, invasiveness, and tumor growth. ↓Self-renewal, tumor-forming capacity, and therapeutic resistance of GSCs	[138]
USP17	Downregulation in glioma. Expression inversely correlated with glioma histological grade. Reduction of RAS and MYC protein levels.	↓Tumorigenesis and proliferation.	[139]
USP26	Stabilization of SMAD7.	↓TGF-β signaling. ↑Survival of patients.	[140]
**Dual role deubiquitinase**			
USP15	Conflicting evidence. USP15 is amplified or deleted in GBM subgroups. It has an oncogenic role by deubiquitinating TGF-βR1 through the suppression of the activity of the ligase complex SMURF2. It exhibits an anticancer role by stabilizing the ligase HECTD1.	↑TGF-β signaling and tumorigenicity (oncogenic). ↑Proliferation and invasion (oncogenic). ↓Wnt signaling (anticancer).	[141–143]

**Table 3:** Compounds with anti-glioma activity through UPS targeting.

Compound	Mechanism of action/Effect	References
**Classic proteasome inhibitors**		
Lactacystin N-acetyl-leu-leu-norleucinal MG132 Proteasome inhibitor II	Activation of the extrinsic and intrinsic apoptotic pathway in glioma cells by suppressing proteasomal degradation of c-MYC, caspase 3, and 8.	[144–146]
Bortezomib	Promotes/reduces the expression of proteins related to cell cycle arrest/progression and pro-apoptotic/anti-apoptotic activity. Exerts a synergistic pro-apoptotic effect with TRAIL. JNK signaling activation. Downregulation of MGMT.	[148, 163–165, 170]
**Synthetic compounds**		
Saquinavir	Inhibits 20S and 26S proteasomes.	[175, 176]
Troglitazone	Sensitizes different GBM cell lines to TRAIL-induced apoptosis via FLIP proteasomal degradation.	[177]
Disulfiram	Inhibition of GSCs proliferation and reduction of glioma development *in vivo*. Disulfiram-Cu complexes suppress proteasome activity and initiate apoptosis in GSCs. Blocks P-glycoprotein extrusion pump activity involved in drug resistance. Promotes changes in the MGMT Cys45 residue, causing its degradation through UPS. The combination of DSF/Cu and TMZ is well tolerated but has limited activity in some patients.	[178–183]
G5	Inhibits isopeptidases. Stimulates necrosis processes in glioma cells resistant to apoptosis.	[184]
LLNle	Proteasome inhibition. Reduction/elevation of genes required for cell cycle progression/suppression. Blocks cell cycle and activates apoptosis in GSCs. Targeting of the NOTCH oncogenic pathway.	[185]
**Natural compounds**		
Geldanamycin	Disruption of HSP90 activity, initiating the ubiquitination and subsequent degradation of proteins interacting with it, including CHK1, CDC2, and cyclin B1. This results in cell cycle arrest, apoptosis onset, and alterations in DNA damage control processes.	[186, 187]
Bufalin	Blocks cell proliferation and initiates apoptosis in GBM cells through proteasomal degradation of ATP1A1. Mitigates tumor growth *in vivo*.	[188, 189]
Thymoquinone	Proteasome inhibition and accumulation of the pro-apoptotic proteins p53 and BAX.	[190]
Hypericin	Stimulates HSP90 proteasomal degradation, causing subsequent lysosomal degradation of HIF-1α (stress-response protein). Causes a reduction in tumor volume and increases survival in GBM patients.	[191, 192]
Paeoniflorin	Inhibits proliferation, activates apoptosis, and suppresses tumor growth *in vitro* and *in vivo* through the promotion of STAT3 and TLR4 proteasomal degradation.	[193, 194]
Sophoridine	Proteasome inhibition, causing significant elevation of ROS levels. Facilitates cell cycle arrest and apoptosis. Inactivates NF-κB.	[195]
Verbascoside	Disrupts cell growth via ubiquitination and proteasomal degradation of c-MET, a protein linked to EMT.	[196, 197]
Curcumin	Synergistic anti-glioma activity in combination with TMZ. Sensitizes GBM cells to TMZ by inducing proteasomal degradation of Connexin 43. The administration of micellar curcuminoids allowed the concentration of significant amounts of Curcumin in GBM patients.	[198, 199]
Obtusaquinone	Activates apoptosis in GBM cell lines and GSCs *in vitro* and *in vivo*. Binds to KEAP1 and reacts with its cysteine residues, inducing its proteasomal degradation.	[200–202]
